# Roadmap for large-scale implementation of point-of-care testing in primary care in Central and Eastern European countries: the Hungarian experience

**DOI:** 10.1017/S1463423622000159

**Published:** 2022-04-21

**Authors:** Csaba Dózsa, Krisztián Horváth, István Cserni, Borbála Cseh

**Affiliations:** 1 Faculty of Healthcare, University of Miskolc, Miskolc, Hungary; 2 Department of Public Health, Semmelweis University Faculty of Medicine, Budapest, Hungary; 3 Cserni Med Bt, Verőce, Hungary; 4 Med-Econ Human Service Ltd, Budapest, Hungary

**Keywords:** Point-of-care testing, POCT, POCT implementation, Primary care, Hungary

## Abstract

**Objective:**

The aim of this study is to give a broad overview of the international best practices regarding the implementation of point-of-care testing (POCT) in primary care (PC) setting and to highlight the facilitators and barriers for widespread national uptake. The study focuses on the managerial and organizational side of POCT, offering a roadmap for implementation as well as highlighting the most important requirements needed to unlock the clinical and economical potential of POCT in the Hungarian healthcare system.

**Methods:**

We conducted an English language scoping literature review between January 2012 and June 2021 to assess the recent trends of POCT implementation in developed countries. Our research focuses on the recent publications of several European and Anglo-Saxon countries where POCT utilization is common. In parallel, we reviewed the Hungarian regulatory framework, ongoing governmental legislation, and strategies influencing the POCT dissemination in the Hungarian PC sector.

**Results:**

Among the possible POCT usage in PC, we identified several clinically relevant devices and tests (C-reactive protein, urine, blood glucose, D-dimer, prothrombin time) important in screening and early detection of morbidities representing high disease burden. Based on international literature, general practitioners (GPs) are interested in the shortened diagnostic times, portable devices, and better doctor–patient relations made possible by POCT. There are several concerns, however, regarding initial and operational costs and reimbursement, limited scientific evidence about quality and safety, unclear regulations on quality validation of tests, as well as managerial aspects like PC staff training and IT integration at the GP level.

**Conclusion:**

As our review highlights, there is considerable interest among GPs to implement POCT as it has the potential to improve quality of care; however, there are many obstacles to overcome before widespread uptake. Further investigation is recommended to elaborate management and quality insurance background and to develop appropriate regulatory framework and financial scheme for GP practices. Preferably this work should involve the local practicing GPs to better tailor the implementation roadmap to country-specific details.

## Introduction

Since the turn of the millennium, the cost and turnaround time (TAT) of diagnostics technology have been decreasing, the devices have shrunk in size, and in many cases became at least as reliable as traditional laboratory equipment. Parallel to these technological innovations, a growing trend of bringing healthcare services, – including but not limited to – diagnostic procedures, as well as more integrated forms of care (community-based care) closer to the population, has been observed. An enabling element of this trend is the emergence and proliferation of point-of-care testing (POCT), which refers to any diagnostic test performed and evaluated at, or near the location of the patient, eliminating the need for the transportation of specimen samples to distant laboratories (Cooke, 2015; Wiencek, 2016). It is safe to say that POCT offers numerous advantages over traditional laboratory testing, both for the patients, the clinicians and health system; however, it must also be stated that there are many obstacles to overcome in a successful POCT implementation strategy in every healthcare system.

These developments translate into the potential of disrupting standard models of diagnostics at various levels of care but are particularly important for general practitioners (GPs) – as diagnostics make up a large portion of the workload in primary care (PC), – lowering costs, enhancing patient experience, strengthening evidence-based clinical decision making, while also decreasing staff workload through eHealth integration, especially in countries with developed healthcare systems (Patel, 2019; Gregory and Lewandrowski, 2009). POCT, which in the Hungarian setting is translated rather as bedside laboratory tests, is utilized by varying degree in the European Union (EU) countries, and Hungary is no exception.

The range of POCT devices and procedures currently utilized in the Hungarian PC setting shows a rather high degree of heterogeneity, with up to 20% of GPs using some form of POCT devices but these early and individual adapters lack key supporting elements: a coherent regulatory background, a professional clinical guideline, as well as reliable quality control or a transparent reimbursement scheme. Parallel to these early users, the need for rapid diagnostics with POCT devices and tests is becoming ever more important, as the number of chronic patients is expected to rise even further given the increasing number of people with multiple (chronic) conditions. Innovations in POCT technology in the recent years increased the range of blood tests and biomarkers available for monitoring serious acute events as well as the status of various chronic conditions (e.g. diabetes mellitus). While POCT is utilized to varying degree in the currently scarcely regulated Hungarian environment, a wide range of challenges (e.g. management, quality control, reimbursement) will need to be universally addressed soon before a large-scale national implementation can begin.

To answer our research questions, we divided our paper into three main parts: (1) we summarized our findings based on the review of international best practices that looked at facilitators and barriers of widespread POCT utilization. Next, (2) we present the clinical applications of POCT by therapeutic areas as well as evidence describing the potential benefits of POCT implementation. Third, (3) we offer proposals for action in the form of a roadmap to overcome the identified difficulties and help in the successful implementation in Hungary.

We believe the proposals formulated here can serve as a practical roadmap for widespread adaptation of POCT devices in Hungary, as well as other similarly developed Central and Eastern European countries and possibly for other middle-income countries globally.

## Methods

Considering the rapid expansion in the volume and type of scientific papers published in the field of POCT in recent years, together with the increasing range and complexity of available tests with clinical applications, we opted to conduct a scoping review of the relevant English language literature between January 2012 and June 2021. The purpose of our scoping review was to provide an overview regarding the identified barriers and facilitators of POCT implementation in the international literature, as well as to summarize the involved managerial challenges focusing on the PC setting. Several systematic reviews covering different POCT clinical applications, GP attitudes, and economic evidence (Lingervelder *et al.,* 2021; Goyder *et al.,* 2020; Jones, 2013) have been published recently, thus we aimed to incorporate a broader overview of the implementation challenges and possible solutions regarding POCT in a PC setting.

In order to help enhance the role of POCT devices in the healthcare technology market, (i) we conducted a scoping literature review on the most relevant international literature describing the possible clinical applications as well as facilitators and barriers of POCT integration in the PC segment, (ii) we carried out interviews with Hungarian Key Opinion Leaders (KOLs), as leading GPs from the PC sector regarding the current legal background and early POCT adaptation experiences. In order to find the most relevant articles, we searched the electronic databases Medline and PubMed using the syntax below:

(“barrier” OR “obstacle” OR “impediment”) AND (“adoption” OR “uptake” OR “acceptance” OR “usage” OR “utilization” OR “utilization” OR “implementation”) AND (‘‘point of care’’ OR “POCT” OR ‘‘near patient’’)

Additional criteria were English language, only Humans, and the availability of full texts which resulted in 1524 results. We screened the results first by their title, and the most promising ones by their abstract, in the end summarizing our findings based on the 34 most relevant articles.

### Summary of international experience

In our review of the international experiences of POCT utilization, we looked at country-specific implementation issues, as well as more general managerial, quality insurance, and financial aspects. We also looked at what kind of organizational changes had to be made in order to successfully integrate POCT into the workflow of GPs and enable the advantages to materialize at the patient care level. We will discuss these potential advantages, as well as factors impeding their achievement, with a focus on the latter as these can serve as the basis for a roadmap aimed at tackling these challenges.

Factors encouraging and hindering the use and spread of POCT devices have been one of the most intensively researched topics in recent years, both in clinical and PC managerial literature (Schols *et al.*, 2018). First of all, it can be stated that the available evidence and experience, – or in many cases, the lack of them –, especially those examining the economic aspects of POCT use, show a nuanced picture. In the case of PC, uncertainties exist among the different therapeutic areas in terms of the realizable efficacy improvements, the reliability of devices, the impact on patient satisfaction and patient pathways, the complexity of training needed to operate near-bed devices, as well as the regulatory and reimbursement framework. Depending on the development stage of these factors, significant differences can be observed between countries, thus influencing the attitudes of local clinicians toward POCT implementation (St John, 2014).

### Facilitators – supporting factors

Summarizing the reviewed literature, the benefits of POCTs are numerous: (i) they enable more effective physician–patient communication; (ii) have the potential to shorten TAT and patient pathways in a meaningful way (enabling an immediate diagnosis thus timely therapeutic decisions without the need for multiple rounds of physician–patient meetings or samples sent to a distant laboratory); (iii) they can improve patient adherence, as POCT results are evaluated “in one sitting”, thus patients become more involved in the therapeutic decision-making, empowering them, and reducing information asymmetry between the parties; (iv) all these factors lead to a better quality of care (Thompson *et al.*, 2013). There was also a general observation about the ease of use of POCT devices both for medical staff and patients (e.g., taking blood from a fingertip, instead of phlebotomy) as reported by Sumita and his research group in their review about different clinical applications of POCT (Sumita *et al.*, 2018).

A clinical trial from the UK demonstrated that in the intervention group utilizing POCT devices, the participating clinicians were able to make therapeutic decisions much faster (74 min) compared to the traditional laboratory-based control group. Recognizing the significance, Larsson *et al.* stated that POCT will “become an integral part of health care management, however expansive quality assurance and training protocols should be established to ensure maximal benefits to patient care and efficiency in any setting” (Larsson *et al*., 2015).

Overall, if POCT can be utilized in patient care, these positive elements are expected to lead to faster decision-making, especially in acute cases (e.g., sepsis) where a rapid diagnosis is paramount for patient safety. POCT tools (e.g. CRP tests) are especially useful in determining the viral or bacterial origin of diseases, thereby effectively reducing unjustified antibiotic prescribing, just as antimicrobial resistance is becoming one of the greatest public health threats in the 21th century. Interestingly, a report reflecting the attitudes of Irish GPs highlighted that a diagnosis based on the results of POCT devices often appeared both as an advantage and a disadvantage from GP’s perspective. This is mostly explained by the lack of evidence being readily available about the accuracy of POCT devices, so in each indication, a negative result is seen as a confirmation of exclusion, while in a positive case, further confirmation is often expected from a sample evaluated by a traditional laboratory (Varzgaliene *et al.,* 2017). This point also highlights the importance of transparent external quality control, as well as the need to address questions about POCT sensitivity and specificity.

Patient satisfaction should also be mentioned as a positive aspect, as patients often report difficulty interpreting traditional laboratory results written in the medical–professional nomenclature, as opposed to the immediately available results of POCT devices, which they can discuss with the physician locally, thereby facilitating better understanding of the diagnosis, strengthening trust, thus enabling further patient education. A review by Crocker *et al.* looked at 97 surveys comparing patient satisfaction between ambulatory practices with or without on-site POCT devices and found that overall patient feedback was very positive where POCT was available (the results showed an average of 3.96 with POCT available, where 4 points was the maximum positive value) (Crocker, 2013).

### Barriers – inhibiting factors

We found numerous factors hindering POCT adaptation during the review of the literature, such as quality control uncertainties surrounding the accuracy, validity (reports with contradictory results) of POCT diagnostics, as well as the scarcity of available trained staff and, most importantly, the cost of equipment and the reimbursement of tests, with further questions about cost-effectiveness raised several times (Varzgaliene *et al.*, 2017). The identified challenges can be categorized into four thematic groups: (1) economic issues, (2) quality assurance and regulatory issues, (3) data management and device performance issues, (4) staffing and training. In our review, we highlight the main issues concerning these topics and offer a possible roadmap (See Figure 3).

The most often cited issues concerning the economic aspects were higher head-to-head cost of POCT compared to traditional testing, while highlighting that their cost effectiveness was difficult to gauge, indicating the need for further research. One of the main challenges mentioned is the longer time scale making the valuation of financial benefits difficult, thus the primary cost justification for POCT utilization is based on the classic “time is money” assumption, hence the rapid TAT, TAT can be translated into benefits in the quality of care (Lingervelder *et al.,* 2021). A further concern relates to the reimbursement structure, as POCT utilization may have a detrimental effect on clinician’s consultation fees, reducing the number of visits, hence making physicians uninterested in utilization. Based on experience from the United Kingdom, Gregory et al proposed the adaptation of diagnostic-related groups financing scheme as a means of controlling the utilization of POCT, in order to avoid unnecessary testing, by rolling up the cost of testing and paying a flat rate for the entire episode of care regardless of how many tests were performed (Gregory, 2009).

For some POCT devices (e.g. HbA1c), the unit cost of the test was found to be higher than the traditional laboratory evaluation (including transportation), but there was a general uncertainty regarding which costs to compare, indicating a high degree of heterogeneity by test types. The perceived higher costs were typically caused by the initial cost of buying the POCT device and the temporal and financial expense of learning/teaching to use it.

Adding to the financial hurdles of wider implementation is potentially the limited economic evidence supporting POCT utilization. A novel systematic literature review examining the health-economic evidence of POCT utilization found however, that majority (70.5%) of the articles focused on PC, and out of 44 studies, only three evaluations found that the benefits of implementing POCT do not outweigh the increase in cost. More than 75% of evaluations concluded that POCT is recommended for implementation, although in some cases only under specific circumstances and conditions. Hence the lack of evidence on POCT effectiveness does not appear to be the primary barrier to its implementation among GPs, there are other inhibiting factors that require further research pinpoint (Lingervelder *et al.,* 2021).

Several studies found that POCT devices are more challenging from a quality control and monitoring perspective when compared with traditional diagnostics methods. Quality issues can occur due to a few reasons, chiefly user errors or the inappropriate storage of reagents, a lack of training, and poor standardization in obtaining blood samples thus insufficient internal/external quality assessment. Quality issues seem to be the main reasons for the underperformance of POCT (Briggs, 2012; Quinn *et al*., 2016). These quality issues with any novel technology are understandable as those working in traditional laboratories view the less well-known innovative POCT diagnostic practices with suspicion, which stems from a lack of experience and general fear of change (Wisse, 2016).

According to a recent study from the Netherlands, the assessment of the additional workload presented by POCT devices in PC – or, in the case of laboratory staff, a decrease – shows a heterogeneous picture: testing staff was typically positive about the new tasks and thus expanding their competence, while physicians were more concerned about the increase in workload. Physician’s main concern was that POCT might generate more promptly available diagnosis possibly leading to the need to treat every patient “on site, immediately”, thus extending the duration of a visit (Verhees *et al.*, 2017).

Integration of POCT into the IT systems used by PC physicians is also a concern to address. Some POCT devices can produce printed results for a patient’s chart, but with paper charts converting to electronic health records (EHRs), results need to be entered manually into a patient’s record, which can be a tedious process. Studies have shown this poses a detrimental effect on the uptake of POCT by the staff, hence the need for POCT devices that’s directly connected to the EHR via electronic interface, which can alleviate some of the staff’s concerns. Interface problem, however, is similar to the different chargers used by mobile phone brands: POCT devices from different manufacturers currently usually utilize their own communication protocols that require company-specific software to communicate results; hence there is an urgent need for regulation and standardization (Wiencek, 2016; Dyhdalo, 2014). We believe these standardized IT systems require regulation to foster change in industry practices.

A recurring concern was the issue of data management, including the question of manual recording and storage of test results, as well as their sharing with the financial agency. It can be stated that POCT can only be successfully implemented by building on well-established, automated data recording (free of human intervention, thus lowering the chance for error) supported with telemedicine solutions integrating GPs IT system with POCT devices, and favorable with the financing agency as well (Verhees *et al.*, 2017). Several GPs stated the lack of IT support as the main cause for not choosing to go ahead with POCT utilization.

Looking at regulatory issues, McNerney *et al.* reported that the complexity of the registration processes required in the UK so far discouraged the necessary up-front investment in the development of POCT devices. A lack of investment in the area directly impacts the potential for the more widespread implementation of POCT devices. The burden of administration should be minimized to the lowest level possible in countries looking to increase POCT utilization (McNerney, 2012). A novel systematic review focusing on European experiences by Seckler *et al.* argues that from a managerial standpoint, in order to overcome the aforementioned difficulties, it is crucial that oversight responsibilities be centralized under the position of a POCT coordinator. The main tasks of this person (preferably just one) should be educating users regarding good laboratory practice and proper operation of all POCT devices, ensuring the maintenance of documentation for POCT-related materials, as well as developing and enforcing adequate quality control procedure for various POCT devices (Seckler, 2020).

In order to get a comprehensive picture, it is essential to mention that one of the major “vulnerabilities” of POCT tools is their utilization without following the proper guidelines (hence the lack of them is a hindrance), which translates to suboptimal or worse, ambiguous results, thus leading to the accumulation of negative experiences and significant material and quality of care losses. The widespread introduction must therefore be preceded by the development of a user guidelines based on professional consensus and evidence-based algorithms followed by dissemination to target users. It is advisable to examine the diagnostic capabilities of POCT in a selected group of GPs, according to a specific indication, during the pilot period. Extending the indication to other patient subpopulations or other conditions may adversely affect diagnostic performance (Nichols *et al.*, 2020). An Irish questionnaire survey among 150 GPs examined the barriers of widespread POCT adaptation and confirmed that the most significant fears were the extra time needed to perform tests in practice, lack of clinical laboratory knowledge, the costs associated with use and maintenance, and questions about accuracy and impact of tests on the therapeutic algorithm (Pai *et al.*, 2012).

In summary, in order to successfully implement a POCT devices in any care settings, several challenges have to be resolved; in our view, the most important are: proper education of staff, a clear regulatory framework, and a reimbursement scheme that does not hurt clinicians’ revenues, transparent quality control program, and a POCT manager in place to manage the diagnostic process. All these components are required for successful implementation, but the most important is probably a well-functioning and easy-to-use IT system which connects the POCT device to the local software applications used by the PC provider.

### Facilitators for and barriers of large-scale POCT implementation

After reviewing the relevant literature, we have summarized the possible facilitators and barriers of large-scale implementation of POCT in PC (See Table 1).

**Table 1.** Facilitators and barriers of the implementation of POCT devices in primary care

	Facilitators for the implementation	Barriers of the implementation
1	Dramatically shortens the diagnosis turnover time, allowing on-site decision-making, and offering time savings for both patient and physician.	There are no domestic care protocols to draw attention to the clinical benefits of POCT that would incentivize GPs to incorporate their use into the structure of day-to-day GP care.
2	Reduced size of the instruments (portability): this allows POCTs to be used next to a patient bed, in an ambulance or in areas away from inpatient facilities even in disadvantaged or more geographically isolated areas.	There is no dedicated asset support and performance-based remuneration in GP financing system. There are no dedicated resources for consumables, validation, depreciation, and quality assurance operation.
3	Reliable and accurate result (with proper quality assurance programs in place).	Successful POCT implementation requires considerable remodeling of the GP’s business model, as well as staff training.
4	They form an important part of the quality assurance (LIS/HIS) system through automated documentation.	The issue of preservation and storage of results.
5	Does not require a complicated laboratory preparation process.	Possible duplication of equipment for laboratory testing (POC and central lab machines).
6	Smaller sample and lower reagent requirements can make POCT solutions less invasive.	Challenges of evaluating and appropriately interpreting results by PC staff.
7	The amount and cost of laboratory work is reduced – resulting in cheaper, more cost-effective tests.	Methodological problems of filtering out erroneous measurement results.
8	Empowerment: patients have a more active role in monitoring their own therapeutic goals, which promotes adherence, while increasing patient satisfaction.	The immediately available “on-hand” testing option may provide an incentive to perform additional tests that are not professionally justified (supply-induced demand).
9	Better outcomes in monitoring chronic diseases, where therapy involves frequent sampling and testing: e.g. C-reactive protein, venereal diseases, acute cardiovascular diseases, deep vein thrombosis, and diabetes.	Initial data management challenges that require robust IT support and eHealth solutions (integrating POCT results automatically into EHR).
10	Economic benefits: although POCT is currently often more expensive than traditional laboratory testing, avoided specialist doctor–patient appointments, lower hospital stays, and fewer hospital referrals can compensate for the initial costs.	The upfront cost of POCT may be prohibitively high and cost of a POCT test may exceed that of a conventional laboratory if the volume of use of the device falls short of the economical level.
11	Explicitly suitable for screening infectious diseases and for determining disease etiology (bacterial or viral), thus helping with antibiotic resistance control.	Burdensome administrative (reporting and documentation) requirements.
12	POCT shows better access and potential in geographically or economically remote areas, with low-quality diagnostic infrastructure, where on-site testing capabilities can most significantly impact the quality and timeliness of care provided.	Lack of harmonization and comparison agreement or contract about test results between central laboratory instruments and POCT devices.

Many of the barriers listed here need to be addressed at the system level, by a radical redesign of the reimbursement, legal background, care protocols, and IT support so that PC can take advantage of the listed benefits and avoid, but at least minimize the disadvantages (Quinn *et al.,* 2016; Kodogiannis, 2014; Ferreira *et al*., 2016).

### Roadmap

Having looked at the general hurdles of POCT implementation, as well as the potential benefits of a successful program, we offer a roadmap based on the gathered insights, highlighting the critical points.

Building on international experience, a crude path can be drawn up for successful POCT implementation in the PC practices. This roadmap of course can vary slightly among countries, hence a “one-size-fits-all” approach is unlikely to work, but the general path is as follows:

- a slow, early adaptation (pilot) phase can be observed, during which the first experiences of POCT use are considered by the actors involved,
- evidence and experiences are slowly generated, as POCT devices gain interest and recognition among the professional bodies and stakeholders,
- draft for guidelines and good practices to be published based on early adapters feedback and insights,
- that will lead to policy and regulatory framework development, and possible wider recognition of the disruptive potential of POCT through dissemination,
- pressure builds on regulators to draft a financial scheme that incentivizes POCT utilization,
- which ultimately paves the way for wider implementation.

It can be observed that in European countries where POCT use is most prevalent at the GP level (specifically the Netherlands, Ireland, and the Scandinavian countries), first adapters who are also KOLs have recognized the diagnostic, therapeutic, and partly the economic benefits of POCT thus “paving” the path for subsequent adapters (Goyder *et al.*, 2020). The cases of Australia and Canada are worth mentioning, where several key cost-effectiveness analyses have been published, and seem to be leading the way in POCT evaluation and evidence generation, hence the systematic spread and use of several POCT device in PC settings can be observed (Wong, et al, 2020; Lingervelder *et al.,* 2021). It might be worth noting that these two geographically large countries are leading in the eHealth field in general, thus the implementation of POCT might have extra incentives due to openness toward innovative healthcare technologies.

A pattern for adaptation can be deduced based on international experiences, where large scale POCT implementation is preceded by targeting a few therapeutic areas presenting significant burden of disease, while at the same time focusing on the range of POCT devices that have a potential for improving treatment outcomes and/or reducing costs. Important aspect to keep in mind, that GPs only feel confident in using a particular POCT device once ample evidence on safety and reliability become available, as well as ease of use and results interpretation is supported (Hardy et al, 2016). Another prerequisite for obtaining benefits from any individual POCT device is the test being sufficient for medical decision-making in the clinical setting, eliminating the need for additional confirmatory tests from the central laboratory.

A potential good example comes from the Netherlands where point-of-care C-reactive protein (CRP) testing is used dominantly to validate the origin of the viral/bacterial infection on-site, which aids in combating a major public health crisis, namely antibiotic resistance, thus understandably an important area for governmental attention and communication (Howick *et al.*, 2016). Because of its importance and public health burden, it is a promising candidate for early POCT integration. Early adopters disseminate experience gained in their clinical practice in the professional sphere as well as continuously generate evidence that can be published to effectively raise awareness for the widespread use of POCT, generating interest and a healthy discussion among stakeholders. As a result of these developments, national organizations can also be informed to start developing guidelines and use cases, as well as to take up and accumulate momentum for the initiative. Through the aforementioned example of fight against antibiotic resistance (which can be communicated efficiently), awareness can be raised to a policy level where macro-level resource allocation decisions can be influenced if enough positive momentum and evidence are gathered, thus allowing the spread of POCT. Based on international experience, this process requires realistically 3–5 years after the first adaptation to mass implementation (Huddy *et al.*, 2016). Experience from the United Kingdom has also shown that early on, GPs need to be incentivized to be willing to integrate POCT into their day-to-day routine. According to European experience, this has encouraged GPs to develop professional guidelines and algorithms for the use of POCT (IFCC, 2014).

**Figure 1.**
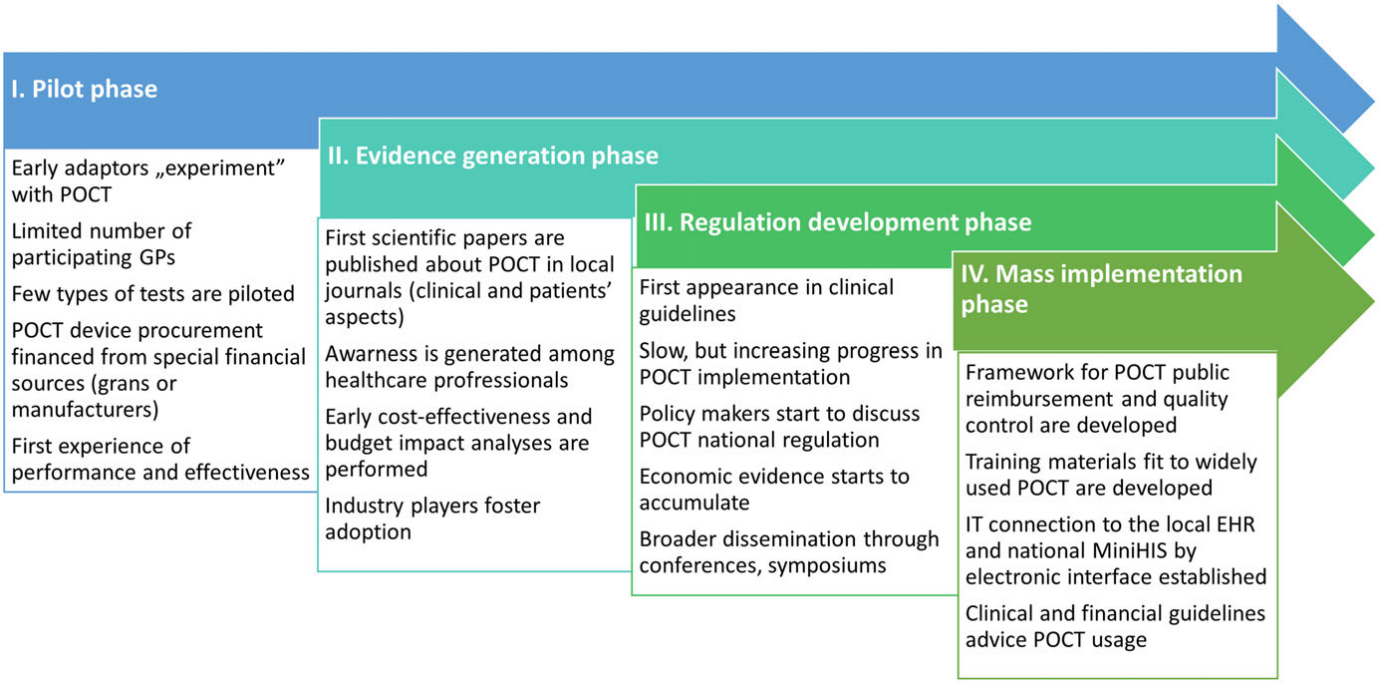
Proposed POCT implementation roadmap in CEE countries.

Based on the gathered insights, we have created a strategic roadmap for POCT adaptation, which can be seen in Figure 1.

### The most important points are shown below:

1) One of the critical points of successful POCT implementation is the development of automated data recording and data transmission, supported by telemedicine solutions, eliminating the chance for human error. As stated in reviews, many GPs expressed a need for comprehensive support in the IT area, with training programs and possibly government funds available to upgrade their hardware and acquire the necessary software (Jones *et al.*, 2013).

2) The second obstacle to overcome is the external quality insurance of POCT. The example from the Netherlands recommends nonprofit central laboratories that can perform this function, as well as the training of staff. Here, the so-called “train the trainer” method can be a good solution, in which the key players take part in training programs, enabling them to pass on their knowledge to colleagues (Kip *et al.*, 2019).

3) From a managerial perspective, there is a clear need for a POCT manager, who oversees the training of personnel, ensures the documentation is in order, might also act as internal quality insurance guide. The manager has to ensure compliance with all applicable national regulations, rules, and standards (Khan, 2019). If these standards do not exist yet, POCT managers should be involved in developing them, as they will possess the most practical knowledge.

4) The fourth (but perhaps most important) point is reimbursement, which must be competitive, so it is proposed to supplement the per capita scheme, often the main source of funding for GPs, with a dedicated POCT financing framework. A good example from England, where CRP testing was included among the financially eligible items through antibacterial care, which is one of the quality indicators of GPs, thus providing a further incentive for POCT utilization (Nichols *et al.*, 2020).

### Clinical application of POCT in general practice in Hungary

In Hungary, several European Union and governmental co-funded projects were carried out between 2012 and 2020, which aimed at strengthening the preventive elements of PC by bringing in novel competencies. To achieve this, the integration of solo PC practices into practice communities was encouraged, improving the collaboration between stakeholders, with the ultimate goal of improving the quality and range of health services provided at community level.

One of the innovative aspects of group practices is the involvement of additional professionals in everyday care such as physiotherapists, dieticians, nurses, psychiatrists, practice nurses, in order to provide more comprehensive preventive services, screening, health assessment, and life-style counseling closer to the patient.

The aim of strengthening GP competencies by delivering additional services closer to the population is to reduce the unnecessary or avoidable attendance on ambulatory care, mainly on specialist visits by providing more extensive (definitive care as much as possible) care at GP level. These pilot programs promote the restructuring of PC, while also opening up avenues for innovative ideas and measures in the provision and organization of publicly funded health care services. The incumbent Hungarian government is showing a continued interest in strengthening PC sector since the mid 2010’s, supported by legislative procedures in 2020–2021, hence we believe the time is ripe to explore the international experience in POCT implementation, and to take proactive measures in formulating strategic plans, that can serve as a roadmap for the local Hungarian setting.

We believe this transition period creates a unique opportunity in Hungary to position POCT, as a cornerstone for a strengthened, prevention, and patient-oriented community-based PC. The professional guideline supports the use of POCT devices and tests, which, along with comprehensive eHealth and quality control background has the potential to shorten diagnostics time, improve patient communication, control costs, and eventually enhance treatment outcomes.

We gathered insights by interviewing several Hungarian GPs about their attitudes regarding POCT. We summarized the main challenges of POCT implementation from the perspective of practicing GPs:

- Low quality of publicly available clinical and health economy evidence, focusing on therapeutic areas with major public health burden;
- POCT utilization is not nationally regulated, and the legal background and professional guidelines have not been developed;
- Reimbursement protocols are not in place, no incentives to deviate from standard laboratory diagnostics, and no budget for initial POCT purchase;
- Quality assurance is inadequate, guidelines (test validation) and safety protocols have to be formulated, and test result documentation regulated;
- eHealth and IT solutions linking GP information system with POCT for data management (and possibly with national healthcare electronic database) are still in infancy;
- Training materials and effective online/offline educational sessions for GP have to be developed and disseminated;
- POCT user manuals need to be adapted to local language.

**Figure 2.**
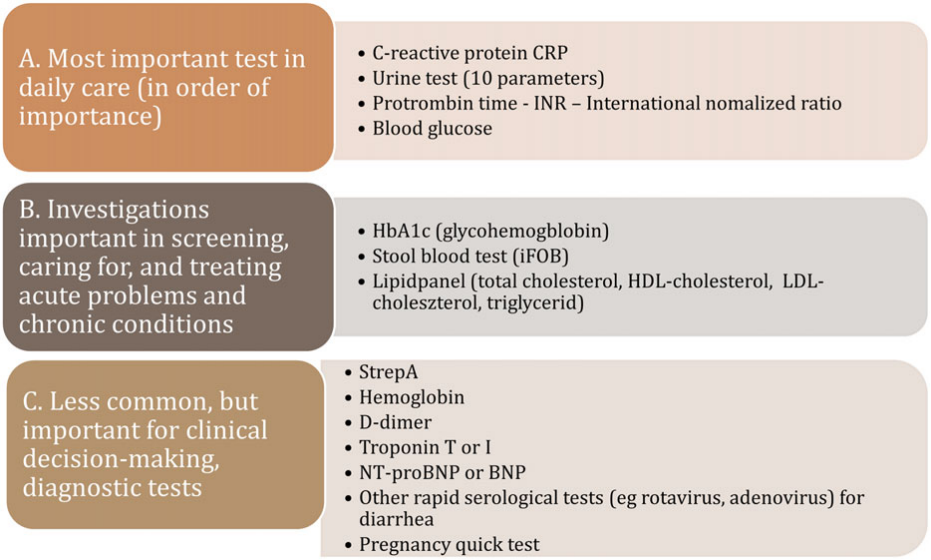
POC tests by frequency of use in primary care.

In the following figure, we classified the most prominent POCT therapeutic areas by their frequency of use: (A) most frequently used test in daily care, (B) regularly used tests utilized in the treating of acute problems of caring chronically ill patients, and (C) rarely used but important in the detection of serious cases (Figure 2).

### Proposals for actions in local environment – example for Hungary

**Figure 3.**
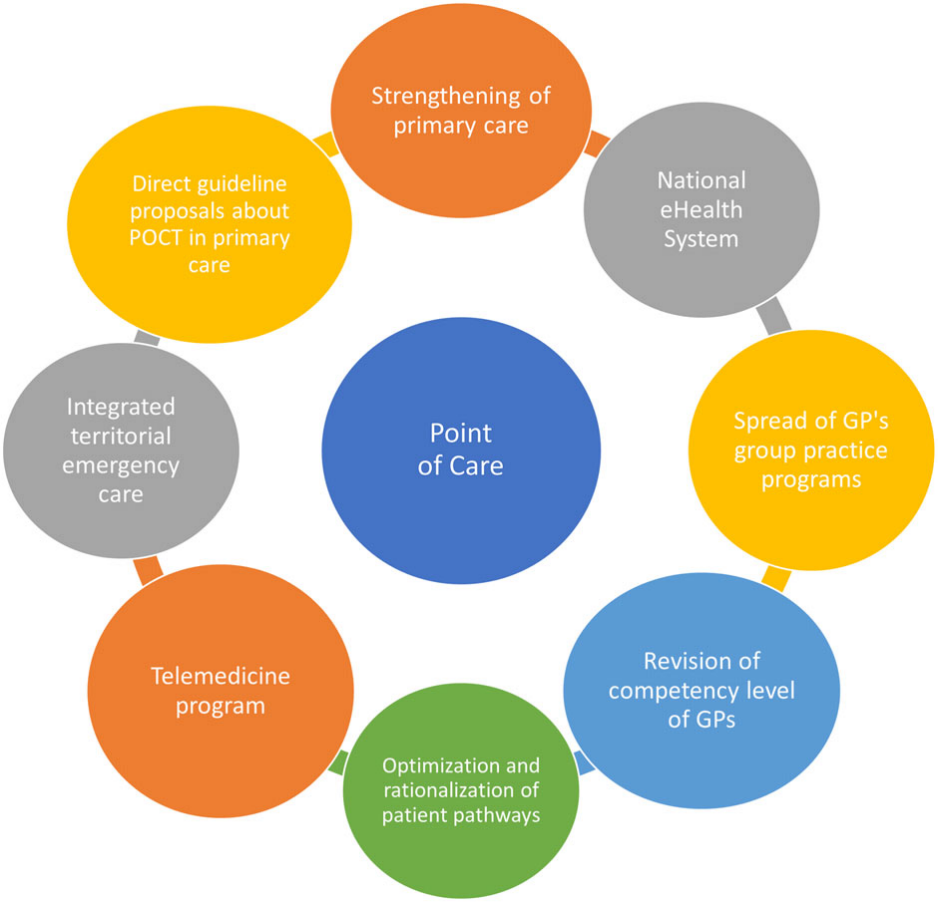
Connecting health system elements, and enabling factors for POCT dissemination in primary care.

Based on the literature, we found that some prerequisites for successful POCT implementation are consistent among countries: continuous training of POCT operating staff, a POCT manager to coordinate administration, introduction of quality assurance (external and internal), telemedicine-supported data management, transparent and incentivizing reimbursement, as well as the generation of ample, high quality clinical and health economy evidence (See Figure 3). We believe that the current Hungarian health policy directions, regulatory, and public financing background is ready to take the next steps for large-scale POCT implementation.

First of all, the widespread utilization of POCT in PC in general fits well to the overall rapidly growing telemedicine program that underwent a significant development during the COVID-19 pandemic. More specifically, it coincides with the goal of providing definitive care and the ongoing enhancement of competency level of the GP practices that are strongly connected to the ongoing practice communities’ (PCO) programs. A freshly released national reform program dictates that the newly established PCs are required to provide several POCT-based activities (e.g. CRP, International Normalised Ratio (INR), troponin, HbA1c measures), and their completion is monitored on a monthly basis (Call - Felhívás, 2021, Annex I). Figure 3 shows the key points in POCT implementation.

A second favorable environmental effect is the ongoing development of the National eHealth System (NEHS) which will soon encompass all healthcare-related data generated by public and private providers in Hungary, thus offering an appropriate information background and digitalized data platform to upload and share the results of POCT tests. An obvious challenge will be the establishment of secure online connection between the various POCT devices and the NEHS, with regular (daily) uploads of test results. Further surveys can be used to identify factors related to the day-to-day use of GP practice as well as higher, system-level influencing factors (regulatory and funding issues).

**Figure 4.**
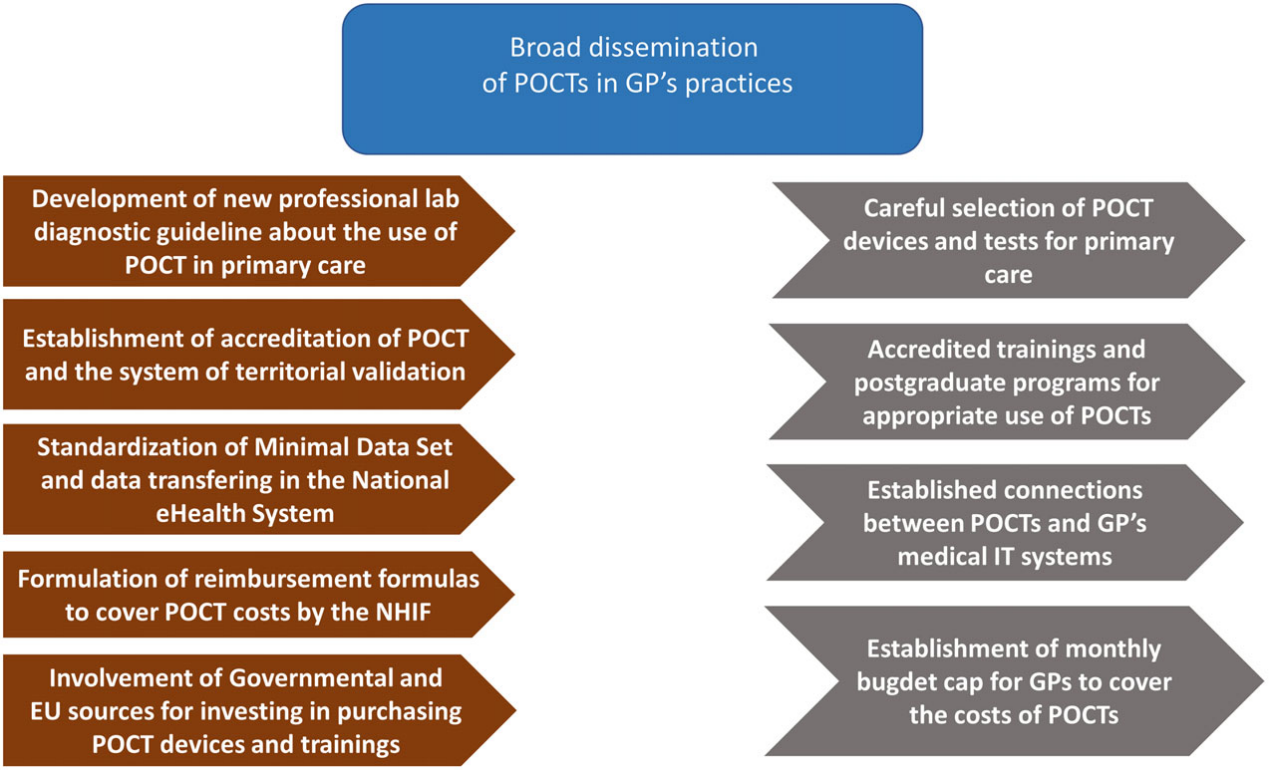
Necessary regulatory framework and interventions in order to support the dissemination of POCTs in the field of primary care.

Our proposals for action are set out below based on the current regulatory and professional policy environment of Hungarian healthcare system, current reform efforts in the field of PC, the legal environment, the referenced opinion of decision-makers at the Ministry of Health and the National Health Insurance Fund, as well as the experiences and evidence gathered via international literature research (See Figure 4). According to our findings, the criteria for the successful widespread implementation and use of POCT devices in Hungary in the community-based care depends mainly on the following factors.

A large-scale qualitative study should be performed among PC physicians to gain information about their current knowledge level and expectations of POCT and use these insights to develop and tailor an implementation plan to local needs. The scope and quality parameters of POCT devices to be used in community-based care (pilot period), including the list of tests, need to be defined, followed by the development of care protocols, professional procedures, and guidelines at a national level. High quality and critical quantity of clinical and health economic evidence in each therapeutic area have to be published. Areas of focus should be: test reliability (sensitivity and specificity), safety of use, and (external) quality control. Economical evidence should support and validate the clinical aspects of POCT. It is also worth noting that POCT users will often be nurses and clinical staff members with existing patient care responsibilities but a limited technical background, thus adequate training or hiring of specialized personnel will most likely be needed, adding extra costs. The available evidence concerning clinical use of POCT has to be gathered and reviewed with the participation of the Professional College of Medical Laboratory Tests.

The technical conditions for the operation of the quality-assured POCT have to be established, and basic issues and templates for contracts with the external laboratories that can serve as external quality controls have to be formulated. Online and offline comprehensive training materials (e-learning and ongoing technical support) have to be developed. Preparation should be in place for a large number of new devices to be procured in the event of a tender, and for a large number of staff to be trained and monitored in a short period of time for a wide range of applications. Dedicated source of funding for GPs has to be allocated and protected by legislation, thus incentivizing the purchase and general use of POCT. This problem is prevalent in Hungary because of the so-called traditional “silo budgeting”, where reimbursement does not follow actual performance, thus eliminating incentives to provide better quality, comprehensive care.

A fair reimbursement scheme for testing must be developed, that can also disincentivize unnecessary/non-guideline utilization, creating a transparent and predictable financing environment. This task has to be undertaken in close collaboration with the National Health Insurance Fund, the sole public payer in Hungary. The financing formula at least has to encompass the following elements: a) the price of consumables, b) the fee for performing additional diagnostic tests, and c) the fee for quality assurance and validation. Monthly budgets could be determined based on previous years (expected number of PC laboratory tests and number of patients, clinical need), for each category of POCT. Definitive care parameters must also be incorporated to incite the gatekeeper function at community level. Framework for automated data recording and data transfer has to be created (telemedicine functions), integrating a POCT device with the IT software of the GP’s practice, ensuring that the validated measurement result is uploaded to the NEHS.

## Conclusions and health policy implications

Given the rate of technological advancement and the potential benefits to efficiency and quality of care offered by POCT, it seems likely that the prevalence of POCT in health care will continue to grow in the future as innovative technologies permit diagnostic tests to leave the confines of the centralized laboratory and migrate to the site of patient care. Timely and reliable diagnosis is the hallmark of effective therapy, and the widespread implementation of POCT devices can make it possible to reduce the burden of disease and mortality presented by diseases. It is reasonable to predict that with POCT use cases targeting the diagnosis of infectious diseases, cardiovascular diseases, and other frequently ordered tests, near-patient testing will become integrated into the PC service provision, thus making POCT a more important part of diagnosis.

Our review of the international POCT utilization experience showed that the primary advantage of POCT devices over traditional laboratory solutions is their speed and ease of sampling for both the patient and the user: POCT eliminates the need for sample transportation likewise the results are typically available in 5–15 min. To achieve these benefits, several key developments have to be made in any healthcare system: a clear regulatory framework, transparent reimbursement scheme, adequate training for the staff, and quality insurance protocols. Coordination with the central laboratory regarding quality assurance and regulatory matters will be crucial as technology allows for a more efficient allocation of testing resources. Likewise, one of the key success factors for the effective use of POCT in PC is the development of a standard IT connection to medical information systems and the national electronic health care system.

To conclude, we believe one of the most important takeaway messages is that shorter TAT provided by POCT allows for accelerated identification and classification of patients by risk groups and increases clinical throughput with a potential to improve quality of care. This benefit, however, can only be realized by the successful reorganization of patient pathways around the POCT devices. POCT might never replace the central laboratory lacking it’s streamlined, high volume “factory-like” testing capacity, rather it can evolve into indispensable component of timely diagnosis, quality patient care in PC setting.
